# The polyphenolic compound punicalagin protects skin fibroblasts from UVA radiation oxidative damage

**DOI:** 10.1016/j.crphar.2024.100186

**Published:** 2024-05-23

**Authors:** Giada Bianchetti, Patrizia Bottoni, Giuseppe Tringali, Giuseppe Maulucci, Elisabetta Tabolacci, Maria Elisabetta Clementi

**Affiliations:** a Dipartimento di Neuroscienze, Sezione di Fisica, Università Cattolica del Sacro Cuore, Largo F. Vito 1, 00168, Rome, Italy; b Fondazione Policlinico Universitario A. Gemelli IRCSS, 00168, Rome, Italy; c Dipartimento di Scienze Biotecnologiche di base, Cliniche Intensivologiche e Perioperatorie, Università Cattolica del Sacro Cuore, Largo F. Vito 1, 00168, Rome, Italy; d Dipartimento di Sicurezza e Bioetica, Sezione di Farmacologia, Università Cattolica del Sacro Cuore, Largo F. Vito 1, 00168, Rome, Italy; e Dipartimento di Scienze della Vita e Sanità Pubblica, Sezione di Medicina Genomica, Università Cattolica del Sacro Cuore, Largo F. Vito 1, 00168, Rome, Italy; f Istituto di Scienze e Tecnologie Chimiche “Giulio Natta” SCITEC-CNR, Largo Francesco Vito 1, 00168, Rome, Italy

**Keywords:** Punicalagin, UVA-Induced damage, Nrf2 pathway, Oxidative stress, Mitochondrial function

## Abstract

Polyphenols are a class of natural compounds that act as antioxidants, neutralising harmful free radicals that would damage cells and increase the risk of diseases such as cancer, diabetes and heart disease. They also reduce inflammation, which is thought to be at the root of many chronic diseases.

We are investigating the photoprotective effects of punicalagin, a type of polyphenolic compound mainly found in pomegranates, against UVA-induced damage in human skin fibroblasts. Punicalagin increases cell viability and reduces the high levels of ROS generated by photooxidative stress through its ability to modulate the Nrf2 transcriptional pathway. Interestingly, activation of the Nrf2 pathway results in an increase in reduced glutathione, NADH, and subsequently protects mitochondrial respiratory capacity. Integrating molecular and imaging approaches, our results demonstrate a potential cytoprotective effect of punicalagin against UVA-induced skin damage through an anti-apoptotic mechanism.

## Highlights

• Punicalagin is a natural polyphenolic compound with a photoprotective effects against UVA-induced damage in human skin cells.
• Its cytoprotective effect against UVA-induced skin damage takes place through a reduction of photooxidative stress.
• The photoprotective properties of punicalagin has the potential to be used as a natural remedy, in food supplements or cosmetic preparations, to protect the skin from the sun, also in the light of issues of environmental sustainability and the circular economy.

## Graphical abstract

**Figure:**
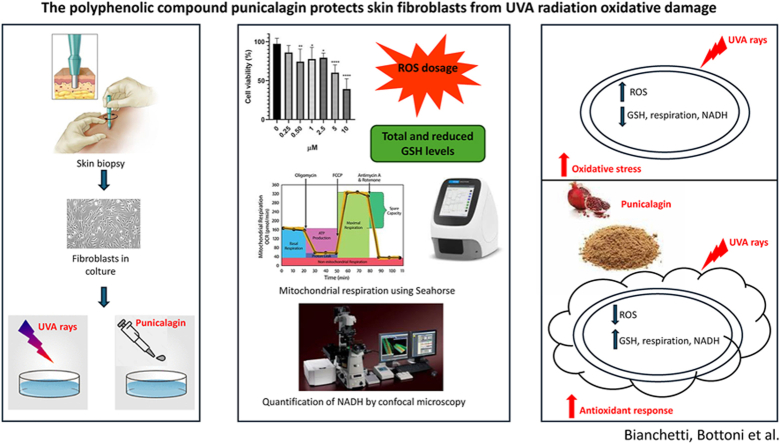

## 1 Introduction

Exposure to sunlight is necessary for human health, e.g. activation of 7-dehydrocholesterol to synthesise vitamin D3 in the skin epidermis to prevent osteomalacia (Wacker and Holick, 2013). However, photodamage induced by UVA and UVB exposure is widely increased, including pigmentation, ageing and even cancer (Battie et al., 2014). UV radiation is classified as a “complete carcinogen”, because it behaves as both a mutagen and a non-specific damaging agent, and has properties of both a tumour initiator and a tumour promoter (D'Orazio, 2013). UV radiation has been epidemiologically and molecularly linked to the three most common types of skin cancer: basal cell carcinoma, squamous cell carcinoma and malignant melanoma, which together affect more than 7 million Europeans each year (Forsea, 2020). Genetic factors may influence the risk of UV-mediated skin diseases, e.g. polymorphisms of the melanocortin 1 receptor (*MC1R*) gene confer increased susceptibility to cancer risk (Manganelli et al., 2021).

Solar UV radiation is divided into three categories based on wavelength: UVA (400-315 nm), UVB (315-280 nm) and UVC (280-100 nm). UVC rays are scattered and reduced by the ozone layer and do not reach the ground. Approximately 90–99% of UVA rays and 1–10% of UVB rays reach the earth's surface. Most of the UVB rays are absorbed by the epidermis. In contrast, UVA rays penetrate deeper into the dermis and their main target cells are keratinocytes, melanocytes and fibroblasts (Tatalovich et al., 2006).

In addition to inducing photodimers of pyrimidines in the genome, UV causes mutations by increasing reactive oxygen species (ROS) such as superoxide anion, hydrogen peroxide and the hydroxyl radical. Nucleotides are highly susceptible to free radical damage, for example by oxidising guanine at position 8 to produce 8-hydroxy-2′-deoxyguanine (8-OHdG), which tends to pair with an adenine instead of a cytosine, causing a mismatch. Normally these mutation events (G/C to A/T) are repaired by Base Excision Repair (BER) mechanisms, but when they accumulate they can be found in skin tumours, suggesting that oxidative damage may be carcinogenic (Agar et al., 2004).

Cells also have several antioxidant molecules that detoxify ROS and prevent free radicals from damaging DNA and other macromolecules. Glutathione (GSH) is one of the most important cellular antioxidant molecules, acting as a reducing agent to neutralise the reactivity of free radicals. GSH is oxidised but can be reduced to its basal state and recycled by glutathione reductase using NADPH as an electron donor. Therefore, GSH can be found in cells in both reduced and oxidised forms, and an abnormal ratio between the reduced and oxidised states is indicative of oxidative stress (Espinosa-Diez et al., 2015). Furthermore, UVA exposure ultimately leads to cellular apoptosis through decreased expression of nuclear factor erythroid 2-related factor 2 (Nrf2) induced by ROS production. Nrf2 is a key factor in protecting skin cells from oxidative stress and UVA radiation (Dinkova-Kostova and Abramov, 2015). In fact, it is a transcription factor that plays a key role in regulating cellular defence mechanisms by initiating a series of cellular responses to oxidative stress and inflammation. Indeed, when cells are exposed to oxidative or inflammatory insults, Nrf2 is activated and translocates from the cytoplasm to the nucleus where it binds to specific DNA sequences known as antioxidant response elements (AREs). This activation leads to the expression of various genes involved in the antioxidant defence system, detoxification processes and other cellular protective mechanisms. Increasing intracellular Nrf2 levels leads to the maintenance of the cellular reducing potential, the reduction of apoptotic genes and the maintenance of mitochondrial integrity, which together improve cell viability. Nrf2 is particularly protective against mitochondrial dysfunction, which can be damaged by ROS production when cells are exposed to oxidative stress. Its activation leads to the expression of antioxidant enzymes and other protective proteins that help to mitigate oxidative stress, particularly within the mitochondria. The main involved enzymes are haem oxygenase-1 (HO-1), superoxide dismutase (SOD) and glutathione peroxidase, which directly protect mitochondria from oxidative damage. Mitochondrial function is protected by the manteinance of both the membrane potential (ΔΨ) and the enzyme complexes involved in the respiratory chain. Nrf2 regulates mitochondrial biogenesis and mitophagy, maintaining the energy balance of cells. Indeed, Nrf2 activation has been shown to control the selective degradation of damaged or dysfunctional mitochondria (Ryšavá et al., 2021; Kahremany et al., 2022). Our focus here is to develop UV protective approaches with a detailed understanding of the molecular events that occur after UV exposure.

In recent years, it has become extremely important to protect the skin from sun exposure with topical sunscreens that form a protective layer against UV radiation on the skin's epidermis. These sunscreens contain UV filters, mostly synthetic molecules that absorb, reflect or scatter UV rays, and are photostable and non-toxic to human cells (Lowe, 2006). Nevertheless, adverse effects of sun exposure on the skin have gradually been observed, suggesting that current commercial sunscreens are not as effective as predicted. Some studies have shown that common UV filters in commercial sunscreens may induce oxidative stress in skin cells due to their photo instability. Thus, additional oxidative damage is added to that which is naturally induced by UV exposure. The additive effect of oxidative events is thought to be responsible for the gradual increase in skin cancer (Rigel, 2002). The detrimental effect of commercial UV filters on ecosystems should also be considered.

Thus, the use of natural compounds to protect against oxidative damage has recently become of emerging benefit and worthy of further implementation (Chen et al., 2016). Here we test the protective effect of punicalagin, a polyphenol, [2,3-(S)-hexahydroxybiphenyl-4,6-(S,S)-gallagyl-d-glucose], which is the major component of pomegranate juice and peel, against UVA irradiation on human skin fibroblasts (Hosseini et al., 2023). Studies have shown that punicalagin may also significantly inhibit oxidative DNA damage and protect against high glucose-induced neural tube defects (Zhong et al., 2015). Polyphenols are also important anti-cancer compounds due to their anti-mutational and anti-proliferative effects. Therefore, several studies have demonstrated anti-cancer activity of punicalagin (Berdowska et al., 2021). It has been reported that punicalagin has several biological effects, including antioxidant (Mohamad et al., 2021), antiviral (Moradi et al., 2019) and antimicrobial (Gosset-Erard et al., 2021) activities, and in particular our recent studies (Clementi et al., 2018a, 2020, 2021) have shown that it can significantly reduce oxidative damage through stimulation of Nrf2, with consequent protection of mitochondrial function, in retinal pigmented epithelial cells exposed to UVA radiation damage. Our study tested punicalagin as a potential protective effector on fibroblasts damaged by UVA radiation, focusing on the role of this molecule in the Nrf2-mitochondria axis. The results obtained are very interesting because they show how punicalagin leads to an increase in Nrf2 and a consequent reduction in UVA-induced mitochondrial dysfunction. Our study aimed at defining the effect of this polyphenol on human dermal fibroblasts exposed to UVA radiation, focusing on oxidative stress and its mechanisms by integrating molecular and imaging approaches.

## 2 Materials and methods

### 2.1 Cells and treatments

A 31-year-old healthy control male (CTRL, coded CTRL1) provided the fibroblasts used in experiments. A fibroblast cell culture was established approximately one week after the biopsy. Informed consent was obtained prior to the skin biopsy, which was performed on the left leg, approximately 10 cm from the ankle (prot. N. 9917/15 and prot. Cm 10/15 of the Ethics Committee of the Catholic University of Rome). The cell culture was grown in DMEM media (Sigma Aldrich, St. Louis, MO, USA) supplemented with 2.5% L-glutamine, 1% penicillin/streptomycin and 10% fetal bovine serum (FBS) at 37° C with 5% CO_2_. Cells were plated at 80% confluence for subsequent studies. A lamp (Vilber Lourmat VL-62C Power 6 W; Vilber Lourmat Deutschland GmbH, Eberhardzell, Germany) set at 365 nm and 10 cm from the source provided UVA exposure with an intensity of approximately 0.06 J/cm2/sec.

Cells were maintained in PBS during exposure to reduce the amount of radiation absorbed by the medium. Immediately after exposure, the culture medium was changed and the cells were left in an incubator for another 24 h before being subjected to the various assays. Under the same experimental conditions, non-exposed cells were kept alive for the same period of time. Based on the cell viability results and our previous study (Tabolacci et al., 2023), we designed the next experiments in which the cells were exposed to UVA radiation for 1 or 2 h. Punicalagin (Sigma-Aldrich, P0023) was dissolved in DMSO to reach a concentration of 10 mM, and the solution was diluted to the appropriate amount in complete medium before use.

Cells were treated with punicalagin 24 h before UVA treatment. We measured cell viability using punicalagin at 5, 10, and 25 μM final concentration after UVA irradiation for 1 and 2 h to evaluate a dose-response curve. The ideal concentration of punicalagin was found to be 10 μM. In addition, 10 μM punicalagin was administered 24 h after UVA exposure. Punicalagin cells that were not treated or exposed to radiation served as controls.

### 2.2 Cell viability

Cell viability was assessed using the MTS assay (Promega srl, Padua, Italy) according to the manufacturer's instructions. Briefly, after punicalagin pretreatment and UVA exposure, and after UVA exposure and punicalagin treatment, MTS reagent was added to the cells and plated in 96-well plates at a cell density of 2 × 10^4^ cells/well until approximately 80% of confluence was reached. The assay provides a sensitive measure of the normal metabolic state of cells, reflecting early changes in cellular redox homeostasis. Intracellular soluble formazan produced by cell reduction of MTS is proportional to the number of live cells and was measured by recording the absorbance of each well of the plate using a plate reader at 490 nm. Cell viability was expressed as a percentage of control cells. Treatment of fibroblasts with [10 μM] punicalagin after UVA irradiation did not increase cell viability, so in the following experiments cells were pretreated with [10 μM] punicalagin and exposed to UVA lamp for 1 and 2 h.

### 2.3 ROS measurement

The amount of intracellular ROS produced was measured using an intracellular ROS detection kit (Abcam, Cambridge, UK) containing 2′,7′-dichlorofluorescein diacetate (DCF-DA). Fibroblasts cultured in 96-well microplates (20,000 cells per well) were treated with DCF-DA according to the manufacturer's instructions and exposed to a variety of experimental conditions. The initially non-fluorescent compound DCF-DA is oxidised to the highly fluorescent chemical DCF in the presence of ROS. Excitation/emission fluorescence at 485/538 nm was measured using a CytoFluor multiwell plate reader (Victor3-Wallac-1420; PerkinElmer, Waltham, MA, USA). Fluorescence intensity was used to measure the amount of ROS generated, which was proportional to the fluorescence released and expressed as a percentage.

### 2.4 Nrf2 detection assay

A cell-based colourimetric ELISA kit was used to measure intracellular levels of Nrf2 (LSBio, LifeSpan Biosciences; Seattle, WA, USA). Fibroblasts were seeded in a 96-well plate at a density of 20,000 cells per well. After UVA exposure and treatment, the cells were fixed with 4% formaldehyde. The plate was then incubated overnight at 4° C with the addition of quenching solution, blocking buffer and primary antibodies (anti-Nrf2-a rabbit polyclonal antibody; anti-GAPDH- a mouse monoclonal antibody, used as an internal positive control and for normalisation). The samples were read using a microplate reader at a wavelength of 450 nm after the addition of the peroxidase-conjugated secondary antibody. The colorimetric results were expressed as a percentage compared to the untreated control and normalised as OD450 of the Nrf2/GAPDH ratio.

### 2.5 Total and reduced glutathione assay

Glutathione levels were measured using a colorimetric assay kit (ab239709, Abcam, Cambridge, UK) to determine total (GSH + GSSG) and reduced glutathione (GSH). In summary, fibroblasts seeded on Petri dishes at 80% confluence were pretreated with 10 μM punicalagin for 24 h, followed by exposure to UVA irradiation for 1–2 h. After irradiation, cells were lysed using the lysis buffer provided in the kit and centrifuged at 14,000×*g* for 10 min. After mg/protein normalisation, total glutathione and the reduced fraction were assayed in the supernatants (see below).

The glutathione assay measures the absorbance at 412 nm to detect the concentration of GSH. This is because glutathione and DTNB (glutathione substrate) react to form 2-nitro-5-thiobenzoic acid, which has a yellow color. Glutathione reductase is omitted from the assay to detect only the reduced form of glutathione. The kit standard curves were used to determine the absolute values of reduced glutathione (μg/μL) and total glutathione (ng/μL). Using a BSA calibration curve, protein concentration was measured in 96-well microplates using a protein assay (Bio-Rad, Hercules, CA, USA).

### 2.6 Mitochondrial purification

Mitochondria were extracted from cells according to the supplier's instructions using a mitochondria/cytosol fractionation kit (MBL, Medical and Biological Laboratories, 200 Dexter Ave., Watertown, MA 02472, USA). Briefly, 5 × 10^6^ cells were incubated for 10 min at 4° C with 1.0 ml of cytosol extraction buffer mix. After homogenization, the cells were centrifuged at 800×*g* for 10 min to extract nuclei and intact cells. The supernatant was collected and centrifuged at 15,000×*g* for 10 min at 4° C. The mitochondrial extraction buffer mix was used to solubilize the pellet fraction. The Thermo ScientificTM PierceTM BCA Protein Assay Kit (Thermo Fisher Scientific, 3747 N. Meridian Road, Rockford, IL 61101, USA) was used to measure protein concentration.

### 2.7 Complex I activity

20 mg of mitochondrial proteins was used to measure mitochondrial respiratory complex I [nicotinamide adenine dinucleotide (NADH)oxidase/coenzyme Q reductase] activity using the MitoCheck kit (Cayman Chemicals; catalog number 700930 Ann Arbor, MI, USA). To ensure that complex III was inhibited, antimycin A (10 mM) was added, and the decrease in NADH oxidation - reflected by a decrease in absorbance at 340 nm - was used to measure mitochondrial complex I activity. The rate of NADH oxidation was monitored for 15 min for each sample. Spectrophotometric measurements of enzyme activity were made in triplicate and results were expressed as changes in absorbance per minute per mg of protein.

### 2.8 Oxygen consumption rate

Oxygen consumption rate (OCR) in adherent cells was measured with the Seahorse XF HS Mini Analyzer using the Seahorse XFp Cell Mito Stress Test Kit (Agilent technologies, Santa Clara, CA, USA). Fibroblasts were seeded in XF HS cell culture microplates at 30,000 cells/well and allowed to grow. The next day, cells were treated with 10 μM punicalagin and, after another 24 h, irradiated with UVA for 1 and 2 h. After irradiation, the growth medium was replaced with Agilent Seahorse XF DMEM medium supplemented with 10 mM glucose, 1 mM pyruvate and 2 mM L-glutamine according to the manufacturer's protocol, and the cells were incubated at 37° C for 30 min to allow temperature and pH equilibration. Control cells were unirradiated and untreated cells that were seeded and allowed to grow for 24 h at 37° C in a 5% CO_2_ humidified incubator, and then placed in Seahorse medium.

After an OCR baseline measurement, 1 μM oligomycin, 0.2–1.0 μM carbonyl cyanide-p-trifluoromethoxyphenylhydrazone (FCCP), 1 μM rotenone, and 1 μM antimycin A were added sequentially to each well. Prior to each experiment, a titration curve with FCCP was performed to determine the optimal FCCP concentration that maximally stimulated respiration. The acidification rate (ECAR) was simultaneously monitored with OCR under different respiration conditions.

### 2.9 Confocal microscopy imaging for NAD(P)H levels assessment

Nicotinamide adenine dinucleotide (NAD) stands out as an intrinsic fluorophore that is widely used in conventional fluorimetric assays, serving not only as a reliable indicator of cellular metabolism (Maulucci et al., 2014), but also as a remarkable antioxidant compound that reflects the cellular antioxidant response (Zipfel et al., 2003; Bianchetti et al., 2022). For NAD(P)H fluorescence microscopy, a Nikon A1-MP confocal microscope equipped with a 2-photon Ti:sapphire laser (Mai Tai, Spectra Physics, Newport Beach, CA) generating 80-fs pulses at an 80 MHz repetition rate was used. A constant temperature of 37° C and 5% CO_2_ was maintained in a stage incubator (OKOLAB S. r.l., Pozzuoli, Italy). Images for different samples were captured using a 60x oil immersion objective (1.4 NA) with a resolution of 1024 × 1024 pixels.

NADH autofluorescence, excited at 740 nm, was recorded in the spectral emission range of 425–475 nm. As NAD(P)H autofluorescence emission correlates with intracellular concentration, fluorescence intensity was quantified using the open-source software ImageJ (NIH) (Bianchetti et al., 2022). The value obtained was then normalised to the number of bright pixels in the image.

### 2.10 Statistical analysis

Each experiment was replicated at least three times with up to eight replicates per group. Results are presented as mean ± SEM. Data were analysed by one-way ANOVA, two-way ANOVA or *t*-test using GraphPad Prism ver. 8 software (San Diego, CA, USA). The significance level was set at p ≤ 0.05.

## 3 Results

**Fig. 1:**
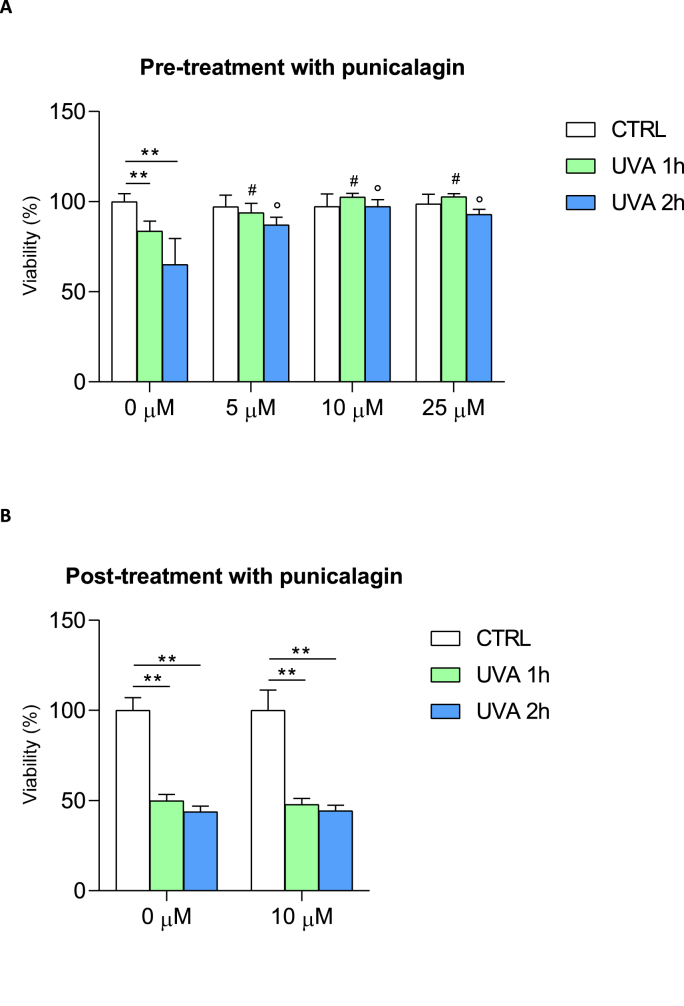
Cell viability in fibroblasts exposed to UVA irradiation and after pre- and post-treatment with different concentrations of punicalagin. (**A**) Cell viability was expressed as % of the control (unexposed and untreated cells). Punicalagin was used at different concentrations (0, 5, 10 and 25 μM). Statistical analysis was performed by comparing data under the same experimental conditions (0, 1 and 2 h UVA exposure). Note the cytoprotective effect of 10 μM punicalagin after 1 and 2 h of UVA exposure. **p < 0.01 (between untreated cells, 0 μM); #p < 0.05 (for 1 h UVA exposure); ° p < 0.05 (for 2 h UVA exposure) by one-way ANOVA (with Geisser-Greenhouse's correction) (**B**) Post-treatment with 10 μM punicalagin did not increase cell viability after UVA exposure (both at 1 and 2 h irradiation). **p < 0.01 (for untreated 0 μM and 10 μM post-treated cells) with *t*-test.

The effect of pre- and post-treatment with punicalagin on cell viability after 1 and 2 h of UVA exposure is shown in Fig. 1. A concentration titration curve was performed at 5, 10 and 25 μM to determine the better concentration of punicalagin pre-treatment. UVA exposure times were 1 and 2 h, based on our previous results (Tabolacci et al., 2023). Thus, cell viability was assessed after treatment of fibroblasts with 5, 10 and 25 μM punicalagin and exposure to UVA irradiation for 1 and 2 h, respectively (Fig. 1a). After UVA exposure, untreated fibroblasts showed a loss of viability of 20 and approximately 40% after 1 (green bar) and 2 (blue bar) hours, respectively, compared to control cells (white bar) that were not exposed to UVA but kept under a hood outside the incubator. Pretreatment with 5, 10 and 25 μM punicalagin had no effect on cell viability in unexposed fibroblasts (compare white bars in Fig. 1a). Punicalagin protected cells from UVA-dependent cytotoxicity even at low concentrations (5 μM), with increases in viability of around 10% and 20% after 1 and 2 h, respectively, compared to UVA-exposed and untreated fibroblasts. This effect was even more pronounced at 10 μM of the compound, with increases in cell viability of 20% and more than 30% at 1 and 2 h, respectively, compared with controls. Similar effects were observed after pretreatment with 25 μM punicalagin. Thus, 10 μM punicalagin was chosen as the lower concentration of the compound with maximum yield. Based on these results, we proceeded to evaluate the effect of post-treatment with 10 μM punicalagin after 1 and 2 h of UVA exposure, respectively (Fig. 1b). Treatment with punicalagin after 1 and 2 h of UVA exposure had no cytoprotective effect.

Collectively, these data demonstrated the protective effect of punicalagin on UVA-exposed cells, and to better identify the molecular mechanisms underlying this result, we performed the following experiments using 10 μM punicalagin prior to UVA exposure.

**Fig. 2:**
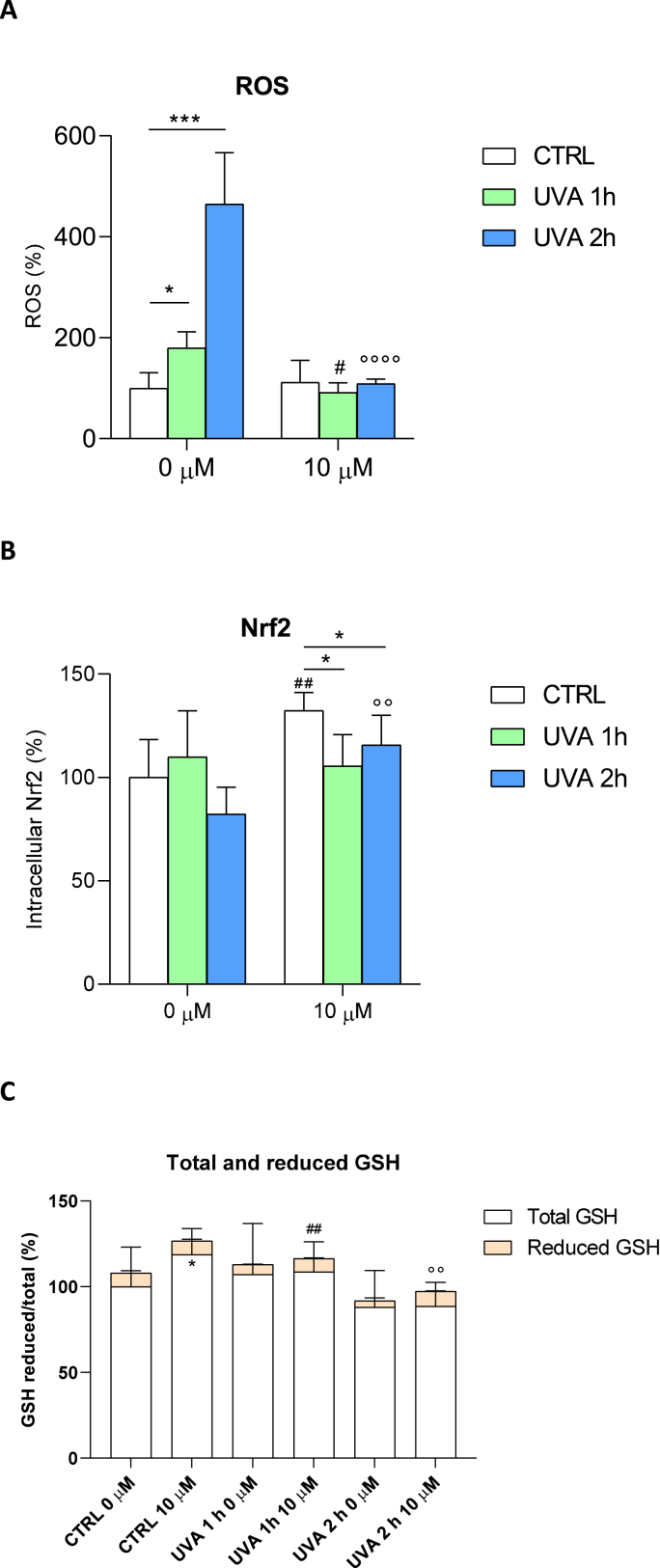
Reactive oxygen species (ROS), Nrf2 and total and reduced glutathione (GSH) levels in fibroblasts unexposed and exposed to UVA for 1 and 2 h, untreated and pretreated with 10 μM punicalagin. (**A**) ROS were expressed as a percentage of fluorescence intensity compared with the unexposed and untreated control, arbitrarily set at 100%. Statistical analysis was performed by comparing data between unexposed vs. exposed and between untreated vs. treated fibroblasts. ROS increased after 1 and even more after 2 h of UVA irradiation in untreated cells, whereas punicalagin rescued these oxidation levels. *p < 0.05 and ***p < 0.001 (for untreated 0 μM cells); #p < 0.05 (for 1 h exposed and pretreated cells); °°°° p < 0.0001 (for 2 h exposed and pretreated cells) with test t (**B**) Nrf2 dosage is presented as percentage of protein compared to untreated and unexposed control (arbitrarily set to 100%). Punicalagin induced an increase in Nrf2 levels in unexposed and exposed fibroblasts, mostly after 2 h of UVA irradiation. *p < 0.05 (for 10 μM pretreated cells) with one-way ANOVA; ##p < 0.01 (control untreated and unexposed vs. control unexposed and pretreated cells);°° p < 0.01 (2 h exposed untreated vs. 2 h exposed and pretreated cells) with *t*-test (**C**) Reduced glutathione is expressed as a fraction of total glutathione and expressed as a percentage in the different experimental conditions. *p < 0.05 (control untreated and unexposed vs. control unexposed and pretreated cells for total GSH); ##p < 0.01 (1 h exposed untreated vs. 1 h exposed and pretreated cells for reduced GSH); °° p < 0.01 (2 h exposed untreated vs. 2 h exposed and pretreated cells for reduced GSH) with test t.

**Fig. 3:**
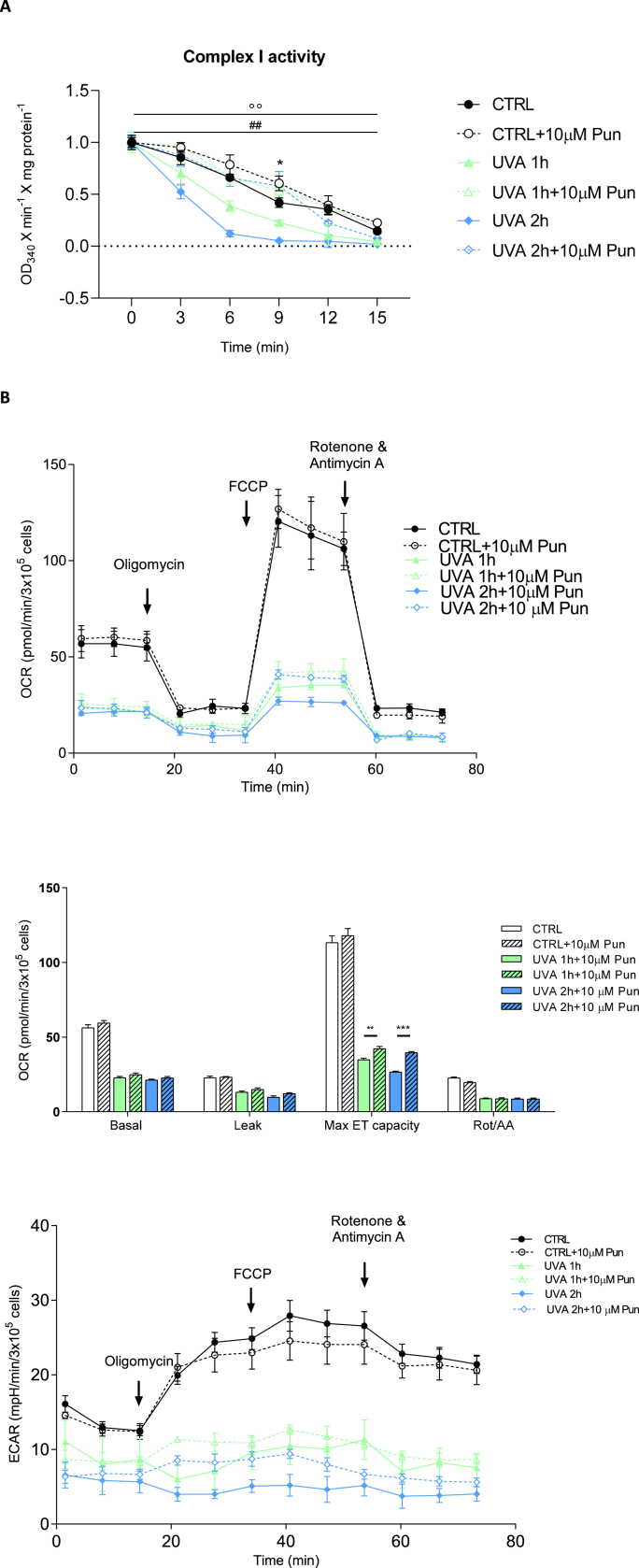
Mitochondrial respiratory chain complexes, function and glycolysis in fibroblasts unexposed and exposed to UVA for 1 and 2 h, untreated and pretreated with 10 μM punicalagin. (**A**) Complex I activity reflects the decrease in absorbance at 340 nm during 15 min of NADH oxidation in the presence of inhibition of complex III by antimycin A. Activity is expressed as absorbance per minute per mg of protein. *p < 0.05 (control untreated and unexposed vs. control unexposed and pretreated cells at 9 min); ##p < 0.01 (1 h exposed untreated vs. 1 h exposed and pretreated cells); °° p < 0.01 (2 h exposed untreated vs. 2 h exposed and pretreated cells) by two-way ANOVA (with Geisser-Greenhouse correction) (**B**) Oxygen consumption rate (OCR) of fibroblasts. OCR is measured before (basal) and after treatment with oligomycin (oligo), carbonyl cyanide-p-(trifluoromethoxy)-phenylhydrazone (FCCP), rotenone (ROT) and antimycin A (AA). A representative OCR measurement of adherent cells in situ is shown (top panel). Quantification of mean OCR ± SEM is shown in the histogram (middle panel). Representative extracellular acidification rate (ECAR) measurements of adherent fibroblasts in the different experimental conditions are expressed as mean ± SEM and shown in the bottom panel. **p < 0.01 (1 h exposed untreated vs. 1 h exposed and pretreated cells after FCCP inhibition); ***p < 0.001 (2 h exposed untreated vs. 2 h exposed and pretreated cells after FCCP inhibition) with *t*-test.

To further investigate the molecular mechanisms of UVA damage and the cytoprotective effect of punicalagin, reactive oxygen species (ROS), Nrf2 and total and reduced glutathione (GSH) levels were measured before and after irradiation and 10 μM punicalagin pretreatment (Fig. 2). First, we quantified the presence of ROS after administration of 10 μM punicalagin followed by UVA irradiation for 1 and 2 h, respectively (Fig. 2a). ROS were measured as fluorescence intensity and expressed as a percentage compared to unirradiated and untreated fibroblasts (arbitrarily set at 100%). In untreated cells, the increase in ROS was doubled after 1 h of exposure and more than quadrupled after 2 h. In contrast, cells pretreated with punicalagin showed oxidation levels similar to those of unexposed cells, with a significant reduction in ROS compared to untreated cells. Thus, punicalagin prevented the increase in oxidation levels caused by UVA irradiation. In addition, increased ROS levels in cells lead to an immediate increase in the production of Nrf2, which attempts to counteract cellular oxidation. With this assumption, we measured this protein in fibroblasts treated according to the established experimental protocol. In Fig. 2b, we report Nrf2 levels in cells unexposed and exposed to UVA irradiation for 1 and 2 h, with and without pre-treatment with 10 μM punicalagin. In fibroblasts not pretreated with punicalagin, Nrf2 levels did not change significantly after 1 h of exposure, whereas a slight decrease occurred after 2 h of UVA irradiation without reaching statistical significance. Pretreatment with punicalagin increased Nrf2 levels and this increase was particularly evident after 2 h of UVA irradiation. One of the mechanisms by which Nrf2 increases cellular antioxidant defences is by enhancing cellular antioxidant potential. We then quantified the levels of glutathione in the cytosol of the cells under our experimental conditions (Fig. 2c). UVA irradiation caused a decrease in total GSH levels, which was rescued by pretreatment with punicalagin. Similar results were obtained for the reduced form of GSH. Table 1 shows data for total glutathione (expressed as ng/μl) and reduced glutathione (expressed as μg/μl). Taken together, these data indicated that irradiated fibroblasts responded by increasing ROS production and depleting cellular antioxidant stores (note the depletion of GSH after 1 and 2 h of UVA irradiation). Pretreatment with punicalagin induced a normalisation of GSH to pre-UVA exposure levels. To better define the protective effect of punicalagin on the respiratory capacity of human fibroblasts, mitochondrial complex I activity and respiratory function were measured in untreated and unexposed cells and with and without pretreatment with punicalagin (Fig. 3). Complex I activity was measured in mitochondria isolated from control, punicalagin-treated, untreated and UVA-exposed cells with and without pretreatment (Fig. 3a). In UVA-treated samples, mitochondrial complex I activity decreased by about 20% after 1 h of exposure and by 55% after 2 h. Pretreatment with punicalagin resulted in the maintenance of complex I enzyme activity similar to that of control mitochondria, again demonstrating a protective effect.

**Table 1:** Total glutathione (expressed as ng/μl) and reduced glutathione (expressed as μg/μl) in fibroblasts untreated (0 μM) and pretreated with 10 μM punicalagin and unexposed and exposed to UVA for 1 and 2 h, respectively. Values were calculated as described in the Materials and Methods section.

	Total glutathione (ng/μl)		Reduced glutathione (μg/μl)	
	0 μM	10 μM	0 μM	10 μM
**CTRL**	68.6 ± 4.3	81.4 ± 5.2*	5.4 ± 0.9	6.5 ± 0.8
**UVA 1h**	73.5 ± 9.0	74.5 ± 2.8	4.0 ± 0.5	5.8 ± 0.3**
**UVA 2h**	60.3 ± 1.6	60.8 ± 5.2	2.4 ± 0.1	5.5 ± 0.2**

*p < 0.05; **p < 0.01 (test t; untreated vs. treated).

Examination of metabolic activity using the Seahorse XF Cell Mito Stress Assay revealed no significant difference in basal mitochondrial oxygen consumption rate (OCR) of punicalagin-treated cells compared to their respective controls (unexposed and UVA-exposed) (Fig. 3b, top and middle panels). Similarly, oligomycin-sensitive respiration, which is induced by inhibition of ATP synthase with oligomycin and corresponds to resting, non-phosphorylating electron transfer, was not affected by punicalagin, suggesting no changes in mitochondrial inner membrane integrity or associated proton transport. However, when UVA-treated cells were stressed by permeabilizing the inner mitochondrial membrane for H^+^ with FCCP to reveal maximal mitochondrial respiratory capacity, pretreatment with punicalagin resulted in a statistically significant increase in OCR of about 17% after 1 h of exposure and 33% after 2 h, confirming a protective role against oxidative damage. Finally, the residual OCR measured after the addition of rotenone and antimycin A, which are inhibitors of complex I and complex III, respectively, showed no perturbation of non-mitochondrial oxygen-consuming processes. The glycolysis activity of the cells was simultaneously assessed as the extracellular acidification rate (ECAR) with the OCR in the same samples (Fig. 3b, bottom panel). There was no significant difference in the ECAR profile between punicalagin-treated cells and the corresponding controls. In UVA-treated samples, after blocking phosphorylation with oligomycin, ECAR induced by punicalagin-treated cells started to increase, indicating activation of glycolysis to restore energy balance.

**Fig. 4:**
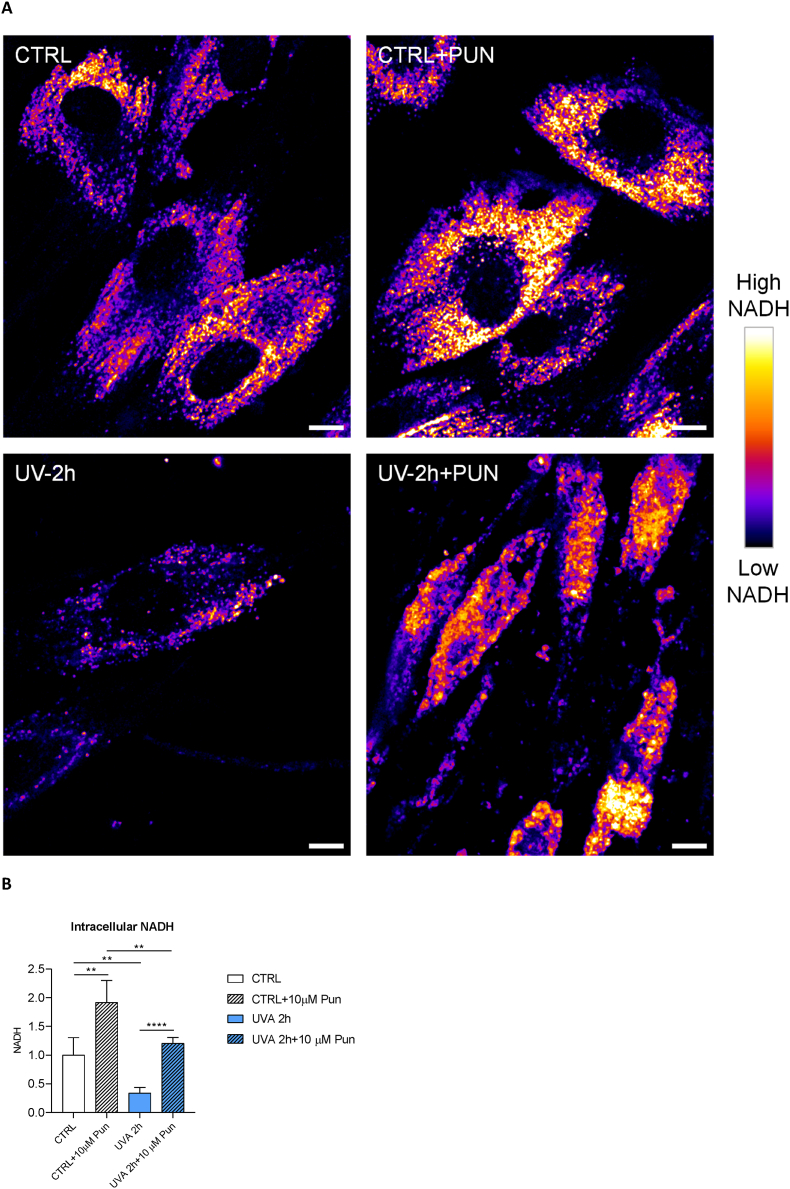
Quantification of NADH by confocal microscopy. (**A**) Representative confocal microscopy images of intracellular NADH autofluorescence for unexposed and 2 h UVA-exposed, untreated and pretreated with 10 μM punicalagin. The four images show maps of intracellular NADH autofluorescence for untreated and unexposed cells (CTRL), treated with punicalagin without UVA irradiation (CTRL + PUN), untreated and exposed to UVA for 2 h (UVA 2 h) and pretreated and exposed to UVA (UVA 2 h + PUN). Pixel colours range from dark purple (low levels of NADH) to yellow (high levels of NADH) according to the color bar reported with the images. The scale bar is 10 μm. (**B**) The bar graph represents the quantification of intracellular levels of NADH, reported as mean ± SD on the y-axis (n = 15 cells). Values were normalised to CTRL (mean NADH = 1.0). **p < 0.01 (control untreated and unexposed vs. control unexposed and pretreated cells; control untreated and unexposed vs. 2 h exposed cells; control unexposed and pretreated vs. 2 h exposed and pretreated cells); ****p < 0.0001 (2 h exposed untreated vs. 2 h exposed and pretreated cells) with *t*-test. (For interpretation of the references to colour in this figure legend, the reader is referred to the Web version of this article.)

Since NADH exhibits autofluorescence, we evaluated its intracellular levels using two-photon microscopy (Fig. 4). Fig. 4a shows the measurement of NAD(P)H autofluorescence using two-photon microscopy. This advanced imaging technique allowed the non-invasive observation of metabolic processes in living cells by detecting the fluorescence of NAD(P)H, a critical coenzyme in cellular respiration. NAD(P)H is fluorescent in its reduced state, whereas it is non-fluorescent in its oxidised state (NAD(P)+). The control image is a baseline image showing the natural state of NAD(P)H autofluorescence. Punicalagin appears to modulate this fluorescence, indicating an increase in NAD(P)H reduction. Instead, an increase in NAD(P)H oxidation is observed in cells exposed to UVA irradiation for 2 h. The stark contrast in the images of cells exposed to UVA irradiation - with and without punicalagin - highlighted the protective or modulatory role of punicalagin against UVA-induced changes. The graph (Fig. 4b) quantifies these observations and provides a clear quantitative analysis of how each treatment alters NADH autofluorescence relative to the control. A significant increase was observed in CTRL + PUN (1.9 ± 0.4) compared to CTRL (1.0 ± 0.3). In contrast, the NADH level in exposed cells (UVA 2 h) decreased to 0.3 ± 0.1, a significantly lower value compared to CTRL. Pretreatment with punicalagin showed the ability to preserve the antioxidant response of fibroblasts exposed to UVA for 2 h, with NADH levels comparable to those of unexposed cells (1.2 ± 0.1).

In conclusion, punicalagin enhanced intracellular NADH production to induce the formation of reactive species.

Taken together, these data suggest a complex interplay between punicalagin, UVA irradiation and cellular metabolic processes.

## 4 Discussion

Fibroblasts are the main cellular components of the dermis and their function is to produce collagen, fibronectin, glycosaminoglycans and other extracellular matrix substances that support the overall structure of the dermis and maintain skin elasticity and hydration. UVA radiation is a type of intense UV radiation that can damage fibroblasts and the extracellular matrix.

Excessive UVA radiation can cause direct damage to skin tissue by attacking proteins, lipids and DNA, and can affect a number of signalling pathways to cause indirect skin damage through ROS-mediated oxidative stress reactions. In addition, to directly oxidising cellular macromolecules such as DNA, proteins and lipids, excessive ROS causes oxidative stress by depleting glutathione and NADPH and altering the redox balance to inhibit redox signalling in cells. In oxidative stress, ROS as second messengers can activate relevant signalling pathways involved in inflammatory damage and tumourigenesis to further regulate downstream gene expression and stimulate various processes such as skin damage, ageing and photo-induced cancer (Sharifi-Rad et al., 2020). Indeed, UVA-induced ROS can promote the phosphorylation of an epidermal growth factor receptor to trigger the mitogen-activated protein kinase (MAPK) cascade response. A large amount of ROS can also trigger the activation of protein kinase-1 (AP-1) and upregulate the gene expression of cyclooxygenase-2 (COX-2), resulting in the secretion of cytokines such as interleukin (IL)-10, IL-8, vascular endothelial factor and prostaglandin G2, which further promote oxidative, inflammatory and necrotic processes (Chen et al., 2021). This process not only accelerates the progression of photo-aging, but also facilitates the proliferation of cancer cells.

The main regulators and transducers of the ROS-induced signal are the mitochondria. Mitochondria are the main site of energy metabolism and ROS production, making them particularly sensitive to the effects of oxidative stress. Indeed, the mitochondrial response to UVA radiation manifests itself in morphological and functional changes leading to loss of respiratory activity, reduced activity of respiratory complexes and irreversible mitochondrial dysfunction (Powers and Murphy, 2019). It is conceivable that the reduction and activity of complex I trigger the increase in ROS, which continue to be produced and accumulated in the cytoplasm until protective antioxidant mechanisms can no longer counteract them.

Under conditions of moderate oxidative stress, cells activate Nrf2 to maintain redox homeostasis. Nrf2 is known to be a master regulator that controls the transcriptional activation of genes involved in antioxidation, antioxidant biosynthesis and metabolic changes (Gado et al., 2023; Lo and Hannink, 2008; Zeb et al., 2021). Often, even this protective mechanism fails in the presence of high levels of ROS. Over the years, the use of molecules, especially natural compounds, with antioxidant activity has proven to be an effective way to counteract the damage caused by oxidative stress. Punicalagin, a polyphenol found in the peel of the pomegranate (*Punica granatum* *L.*), is a natural compound capable of exerting anti-inflammatory, cardioprotective, neuroprotective, wound healing, antimicrobial and anticancer properties. This bioactive compound develops its multiple pharmacological effects mainly through its antioxidant activity (Singh et al., 2023). In a recent paper, punicalagin was delivered to the mouse brain using an intracranially injectable therapeutic hydrogel (Ju et al., 2023). The authors demonstrated the efficacy of the polyphenol therapeutically encapsulated injectable hydrogels on neuronal regeneration and demonstrated the potential of this new type of antioxidant biomaterial in the therapy of brain disorders. Punicalagin possesses antioxidant activity with potential application to protect the skin against UVA radiation damage. Its photoprotective activity is strongly dependent on its ability to reach the viable skin layers. Using an *in vitro* model, the efficacy of nanoemulsions to deliver the polyphenol punicalagin to deeper skin layers has been demonstrated, paving the way for its application in sunscreens (Baccarin and Lemos-Senna, 2017).

We exposed human fibroblasts to UVA radiation to obtain an *in vitro* model of photo-induced damage. UV irradiation triggers a series of chain reactions that initially determine an increase in intracellular ROS levels, which favour apoptotic processes and consequently cell death. Our previous work, carried out on different cellular models and on the same cell type, shows that the increase in ROS levels resulting from photo-oxidative damage is linked to the activation of the signalling pathways of the transcription factor Nrf2, leading to apoptosis (Clementi et al., 2020, 2022; Tabolacci et al., 2023; Bianchetti et al., 2022).

Interestingly, the observed increase in ROS after UVA exposure is not followed by an increase in Nrf2 levels. Normally, the pro-oxidative stimuli increase ROS levels, leading to a ready activation of the Nrf2 pathway, but in the case of UVA irradiation, the increase in ROS levels did not lead to an immediate increase in Nrf2 levels. These results were consistent with our previous observations (Clementi et al., 2018b, 2020; Tringali et al., 2016) and others in the literature (Ryšavá et al., 2021). We hypothesised that the cellular response and nuclear translocation of Nrf2 follow different times and modes depending on the chemical and physical properties of the noxious stimuli. Based on our results, it can be speculated that GSH, the main cellular antioxidant defender, directly interacts with ROS to form oxidised glutathione (GSSG) and that the regeneration of oxidised GSH (GSSG) is mediated by an enzyme, glutathione reductase (GSR), which is stimulated by Nrf2. We observed a decrease in reduced GSH upon UVA exposure, a sign of an immediate attempt by the cells to defend themselves against oxidative stress.

Pretreatment with punicalgin predisposes cells to respond to UVA-induced photo-oxidative damage with a consequent increase in ROS, as Nrf2 is already elevated in unexposed cells. This increase also leads to a greater capacity of cells to respond to oxidative stress in the short term, as indicated by reduced GSH levels. The increase in Nrf2 thus endows cells with an antioxidant potential that facilitates the scavenging of ROS after an acute insult such as that produced by UVA exposure.

It is important to note that punicalagin (both α and β isomers) is able to induce cell cycle arrest at the S-phase and an increase in DNA fragmentation, which are indicators of the induction of apoptosis (Tamborlin et al., 2020).

To evaluate the ability of punicalagin to counteract oxidative damage, we pre-treated fibroblasts with 10 μM concentration of punicalagin for 24 h, then exposed the cells to UVA for 1 and 2 h and tested their mitochondrial function. Punicalagin did not rescue the basal OCR of UVA-exposed cells, but it is able to significantly restore the loss of maximal OCR displayed by fibroblasts subjected to UVA-induced oxidative stress. In UVA-exposed samples, maximal OCR, which is a measure of mitochondrial respiratory system functionality and is independent of cellular energy demand, was significantly increased after pretreatment with punicalagin compared to control. At basal levels, UVA-exposed cells were operating closer to maximum OCR capacity and any increase was unsustainable, resulting in a lower reserve capacity compared to their pre-treated counterparts. Measurement of OCR is very useful in assessing the functional properties of mitochondria. In particular, this method, which allows the assessment of respiratory function without the addition of exogenous substrates and ADP, has been shown to be particularly suitable for preserving all mitochondrial perturbations affecting cellular respiration (Brand and Nicholls, 2011; Bottoni et al., 2022). Interestingly, these results are consistent with measurements of complex I activity. Respiratory complex I is a key player in the way organisms obtain energy, being an energy transducer that couples nicotinamide adenine dinucleotide (NADH)/quinone oxidoreduction with proton translocation by a mechanism that remains unexplained. The reduction in complex I activity following UVA exposure, which is reversed by pretreatment with punicalagin, is therefore essential for maintaining the redox potential of cells.

Taken together, these results clearly show that fibroblasts pre-treated with punicalagin have a higher adaptability to stress and mitochondrial ability to meet a sudden demand for additional energy, demonstrating a protective role for punicalagin against oxidative damage.

Furthermore, the advanced non-invasive imaging approach used to detect the (auto)fluorescence of NAD(P)H in living fibroblasts suggests a protective mechanism at the cellular level that may be critical for skin health. By altering the redox state of cells, punicalagin appears to mitigate UVA-induced oxidative stress. For a more detailed exploration of the interplay between NADH and glutathione, it is crucial to understand how these two key antioxidants interact and influence cellular responses to oxidative stress. NADH, a coenzyme involved in redox reactions, plays a critical role in cellular energy metabolism and oxidative stress response. Glutathione, another essential antioxidant, works synergistically with NADH to maintain cellular redox balance. This interplay is particularly important under UVA exposure where oxidative stress is increased. NADPH and GSH are closely linked in cellular defence against oxidative stress. NADPH acts as a vital electron donor in the reduction of oxidised glutathione (GSSG) back to its reduced form (GSH). This process is essential for maintaining cellular redox balance. During oxidative stress, glutathione is oxidised while reactive oxygen species (ROS) are neutralised, and NADPH plays a key role in regenerating reduced glutathione, thereby maintaining the cell's ability to counteract oxidative damage. The modulatory effects of punicalagin on NADH and possibly GSH levels may suggest a dual mechanism for combating oxidative damage. Investigating how these pathways are affected by punicalagin may provide deeper insights into its protective role and pave the way for targeted therapeutic strategies in skin care and disease prevention. The study also contributes to the growing body of research on natural compounds in photoprotection, highlighting the importance of understanding cellular responses to environmental stressors. Future research could explore the molecular pathways involved in this protective mechanism, potentially leading to new strategies to prevent UV-induced skin damage.

In conclusion, the present study extends our previous knowledge of the nutraceutical properties of punicalagin. Although our data require further analysis and validation, they demonstrate that punicalagin is a polyphenolic compound with high photoprotective properties, capable of protecting skin fibroblasts from UVA-induced oxidative damage via the Nrf2 signalling pathway. Nrf2 is a transcription factor that regulates the gene expression of several antioxidant proteins. Therefore, based on our results, it can be hypothesised that punicalagin has the potential to be used in the development of innovative functional foods, nutraceuticals and other value-added products, providing new opportunities for the pharmaceutical, cosmetic and food industries.

## Funding

This study was funded from Fondazione Policlinico Universitario ‘A. Gemelli’ IRCCS “Ricerca Corrente 2023” (to ET and GT).

## Informed consent statement

Informed consent was obtained from individual involved in this study.

## Institutional review board statement

The study was conducted in accordance with the Declaration of Helsinki, and approved by the Ethics Committee of Catholic University (prot. N. 9917/15 and prot. cm 10/15, April 8, 2021) to perform human skin biopsies.

## CRediT authorship contribution statement

**Giada Bianchetti:** Methodology, Formal analysis, Investigation, Writing – original draft, preparation. **Patrizia Bottoni:** Methodology, Formal analysis, Investigation, Writing – original draft, preparation. **Giuseppe Tringali:** Formal analysis, Data curation, Writing – review & editing. **Giuseppe Maulucci:** Methodology, Formal analysis, Data curation, Writing – review & editing. **Elisabetta Tabolacci:** Conceptualization, Methodology, Investigation, Data curation, Writing – original draft, preparation, Writing – review & editing. **Maria Elisabetta Clementi:** Conceptualization, Methodology, Investigation, Data curation, Writing – original draft, preparation, Writing – review & editing. All authors have read and agreed to the submitted version of the manuscript.

## Declaration of generative AI and AI-assisted technologies in the writing process

During the preparation of this work the authors used MS Word text prediction in order to check grammar and language. After using this service, the authors reviewed and edited the content as needed and take full responsibility for the content of the publication.

## Declaration of competing interest

The authors declare the following financial interests/personal relationships which may be considered as potential competing interests:

Elisabetta Tabolacci reports administrative support and equipment, drugs, or supplies were provided by University Hospital Agostino Gemelli. Elisabetta Tabolacci reports a relationship with Catholic University of the Sacred Heart that includes: employment. If there are other authors, they declare that they have no known competing financial interests or personal relationships that could have appeared to influence the work reported in this paper.

## Data Availability

Data are available within the article
